# Effects of photobiomodulation on stem cells from human exfoliated deciduous teeth cultured on electrospun polylactic acid microfibrous scaffolds

**DOI:** 10.1007/s10103-026-05049-2

**Published:** 2026-09-29

**Authors:** Vladimir Galdino Sabino, Fernanda Ginani, Talita da Silva Grimke, Carlos Eduardo Bezerra de Moura, Hugo Alexandre de Oliveira Rocha, Paulo Henrique de Souza Picciani, Carlos Augusto Galvão Barboza

**Affiliations:** 1 Department of Biochemistry, Federal University of Rio Grande do Norte, Natal, Brazil; 2 Department of Morphology, Federal University of Rio Grande do Norte, Natal, Brazil; 3 Instituto de Macromoléculas Heloisa Mano, Federal University of Rio de Janeiro, Rio de Janeiro, Brazil; 4 Department of Animal Sciences, Federal Rural University of Semiarid Region (UFERSA), Mossoró, Brazil; 5 INCT-BIOFOTO BUCAL, National Institute of Science and Technology in Translational Biophotonics in Dentistry, Porto Alegre, Brazil

**Keywords:** Photobiomodulation, Stem cells from human exfoliated deciduous teeth, Polylactic acid, Electrospun scaffolds, Cell metabolic activity, Tissue engineering

## Abstract

This study aimed to evaluate the short-term effects of photobiomodulation (PBM) on stem cells from human exfoliated deciduous teeth (SHEDs) cultured on electrospun polylactic acid (PLA) microfibrous scaffolds. The scaffolds were characterized by scanning electron microscopy, X-ray diffraction, thermogravimetric analysis, and differential scanning calorimetry. SHEDs were seeded onto the scaffold surface and assigned to three groups: nonirradiated control (C), L0.5 (0.5 J/cm²), and L1 (1.0 J/cm²). PBM was delivered with a 660 nm diode laser at 0 and 48 h after the initial irradiation time point. Cell metabolic activity was assessed at 24, 48, and 72 h using the Alamar Blue assay. Qualitative viable-cell distribution and cell–scaffold interactions were evaluated by Live/Dead staining and scanning electron microscopy, respectively. SHEDs remained viable on the PLA microfibrous scaffold and showed increasing metabolic activity over time. Compared with the control group, L1 presented higher Alamar Blue reduction values at 48 and 72 h, with the highest metabolic activity observed at 72 h. Live/Dead images showed qualitatively more extensive viable-cell colonization of the scaffold surface in the irradiated groups, without an apparent increase in dead-cell staining. Scanning electron microscopy revealed SHED adhesion to the microfibrous scaffold in all groups and qualitatively more extensive cell coverage after irradiation, particularly in L1. Electrospun PLA microfibrous scaffolds supported short-term SHED culture, and PBM at 1.0 J/cm² increased SHED metabolic activity and was associated with favorable qualitative cell–scaffold interactions. These findings provide preliminary support for PBM as an adjunctive biophysical strategy for improving early cellular responses in SHED-seeded electrospun PLA scaffolds.

**Graphical Abstract:**

**Figure:**
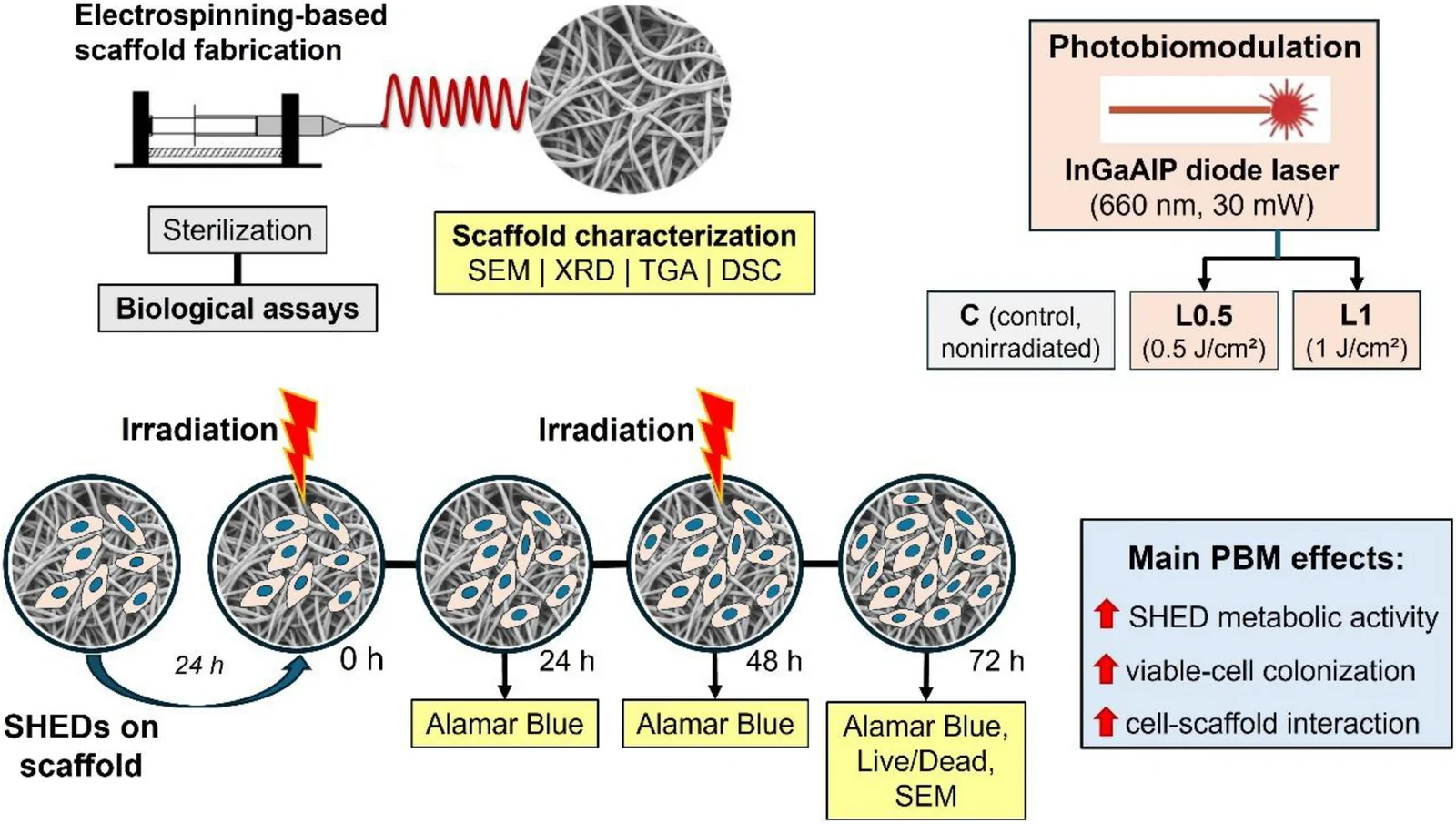
Electrospun PLA microfibrous scaffolds were fabricated, sterilized, and characterized by SEM, XRD, TGA, and DSC. SHEDs were seeded onto the scaffolds and exposed to photobiomodulation with a 660 nm InGaAlP diode laser immediately after the 24-h adhesion period and again 48 h later in the irradiated groups (L0.5 and L1), whereas the control group remained nonirradiated. PBM at 1.0 J/cm² increased SHED metabolic activity and was associated with qualitatively more extensive viable-cell colonization and favorable cell–scaffold interactions at 72 h.

## Introduction

Tissue engineering aims to generate new tissues for the reconstruction or replacement of damaged structures through the integration of three essential components: a scaffold that provides structural support, a cell source capable of forming the target tissue, and biochemical or biophysical cues that regulate cell behavior within the construct [1, 2]. In this context, scaffolds play a central role in tissue engineering because they are designed to mimic key structural and functional features of native tissues and their extracellular matrix (ECM), thereby providing a microenvironment that supports cell behavior [3]. Their performance depends not only on their composition but also on the optimization of their structure and mechanical properties, which directly influence adhesion, proliferation, differentiation, and migration [4].

Among the different scaffold architectures, polymeric fibrous scaffolds are particularly attractive because of their high porosity and large surface area, both of which favor cell–material interactions. These structures can be produced by several techniques, among which electrospinning has been widely explored because it enables the fabrication of ultrafine fibers ranging from the micro- to nanoscale [5]. Electrospun fibrous scaffolds are regarded as suitable artificial ECM analogs because their porous architecture and high surface-to-volume ratio provide a favorable interface for cell attachment and support mass transport within the construct [4]. Among the biodegradable and biocompatible polymers used in this technique, polylactic acid (PLA) is among the most widely investigated because of its biocompatibility, biodegradability, mechanical strength, relatively low cost, and established biomedical use [6]. Electrospun PLA fibrous membranes can therefore be used as scaffold platforms for tissue engineering applications, as they provide an ECM-like topography that favors cell attachment and may also support surface biofunctionalization and the delivery of bioactive molecules [7].

The development of in vitro tissue-engineered constructs also requires a cell source capable of adhering to and populating the scaffold surface and contributing to the functional properties of the target tissue [8]. In this context, stem cells are particularly attractive because of their self-renewal capacity and multipotent differentiation potential. Stem cells from human exfoliated deciduous teeth (SHEDs), which are derived from the embryonic neural crest, have been widely investigated in regenerative medicine and tissue engineering because of their high proliferative capacity and multilineage differentiation potential [9, 10].

Photobiomodulation (PBM) has emerged as a promising adjunctive strategy in tissue engineering because of its ability to modulate cellular responses in vitro, particularly in stem cells. Previous evidence has highlighted the potential of low-level laser irradiation in stem cell-based tissue engineering and has shown that PBM can positively influence SHED proliferation in vitro [9]. In this context, PBM has been proposed as a biophysical stimulus capable of complementing the classical tissue engineering triad of cells, scaffolds, and signaling molecules, further supporting its relevance as a noninvasive approach for regenerative applications [11, 12]. The beneficial effects of PBM have also been reported in cells cultured on PLA-based biomaterials, including MC3T3-E1 preosteoblastic cells on PLA films [13] and periodontal ligament stem cells on PLA scaffolds [14]. Together, these findings support the use of PBM as a strategy to improve the biological performance of stem cells cultured on electrospun PLA scaffolds. From an application perspective, SHED-seeded electrospun PLA microfibrous membranes may be explored as scaffold-based platforms for oral and craniofacial tissue engineering strategies, particularly in approaches in which early cell adhesion, survival, and expansion on a biomaterial surface are needed before subsequent differentiation or functional maturation.

Although the biological effects of PBM have been widely documented, the molecular mechanisms underlying these responses remain only partially understood. At the cellular level, PBM has been associated with increased mitochondrial activity and ATP production, which may contribute to enhanced cell proliferation [15, 16]. In addition, the absorption of light by endogenous chromophores can trigger intracellular signaling pathways that regulate the expression of genes involved in cell proliferation, migration, anti-inflammatory responses, antiapoptotic pathways, and antioxidant activity [12, 17].

Based on this rationale, we hypothesized that combining the biological effects of PBM with the structural features of electrospun PLA scaffolds could improve early SHED responses in vitro. Therefore, the present study aimed to evaluate the short-term effects of PBM delivered by a 660 nm diode laser on the metabolic activity, viable-cell distribution, and qualitative cell–scaffold interactions of SHEDs cultured on electrospun PLA microfibrous scaffolds.

## Methods

### Materials and scaffold fabrication

Dimethylformamide (DMF) and poly(lactic acid) (PLA; molecular weight 93,100 Da) were purchased from Sigma‒Aldrich (Brazil). PLA microfibrous scaffolds were produced by electrospinning from a solution of 16% PLA (g·mL^− 1^) in chloroform/DMF (8:2, v/v) at a flow rate of 0.5 mL·h^− 1^, an applied voltage of 20 kV, and a needle-to-collector distance of 13 cm using a horizontal setup. The temperature and relative humidity were monitored but not controlled during the process. Electrospinning was carried out for approximately 3–4 h to obtain the PLA scaffolds.

### Characterization of the electrospun fibers

Fiber morphology was evaluated by scanning electron microscopy (SEM; JEOL JSM-5300). The samples were then sputter-coated with gold for 2 min and examined at an accelerating voltage of 20 kV. The fiber diameter was measured using Size Meter^®^ software. X-ray diffraction (XRD) analysis was carried out over a 2θ range of 2–50° with a step size of 0.02°. The degree of crystallinity was calculated by integrating the relative peak areas using Fityk^®^ software. Thermogravimetric analysis (TGA) and differential scanning calorimetry (DSC) were performed on both the electrospun PLA fibers and unprocessed PLA under a nitrogen atmosphere. TGA was conducted from 30 °C to 700 °C at 10 °C/min and a nitrogen flow rate of 40 mL/min. DSC was performed from − 10 °C to 100 °C at 10 °C/min.

### Cell culture

The experiments were performed using stem cells from human exfoliated deciduous teeth (SHEDs) derived from a single donor that were previously isolated and characterized as described elsewhere [9]. The cells were cryopreserved in fetal bovine serum (FBS) supplemented with 10% dimethyl sulfoxide (DMSO). This study was approved by the Research Ethics Committee of the Federal University of Rio Grande do Norte (Approval No. 34913314.4.0000.5537) and was conducted in accordance with the ethical standards of the Declaration of Helsinki. After being thawed, the SHEDs were cultured in α-MEM supplemented with 15% FBS (Gibco, USA) at 37 °C in a humidified atmosphere with 5% CO₂, and the medium was changed every 3 days. At passage 4 (P4), the cells were recharacterized by plastic adherence, surface marker expression (CD105, CD73, and CD90 positive; CD45, CD34, CD11b, CD19, and HLA-DR negative) by flow cytometry using the Human MSC Analysis Kit (BD Stemflow™, USA), and osteogenic and adipogenic differentiation using commercial kits (StemPro Differentiation Kits, Invitrogen, USA) over 21 days.

### Scaffold preparation and cell seeding

Electrospun PLA scaffolds were cut into 6-mm-diameter discs and sterilized according to Mansourizadeh et al. [18] by UV exposure for 3 h in a laminar flow cabinet, immersion in 70% ethanol for 30 min, and three washes in PBS for 15 min each. The scaffolds were then placed in 96-well plates and preincubated overnight in α-MEM supplemented with 15% FBS at 37 °C and 5% CO₂ to promote wetting before cell seeding. SHEDs at passage 5 (P5) were seeded onto the scaffolds at a density of 5 × 10³ cells/well and incubated for 24 h to allow cell attachment. The samples were then assigned to three groups: nonirradiated control (C), 0.5 J/cm² (L0.5), and 1.0 J/cm² (L1).

### Photobiomodulation protocol

Photobiomodulation was delivered using an InGaAlP diode laser (Kondortech, Brazil) emitting at 660 nm in continuous-wave mode. Laser output power was measured using a LaserCheck Power Meter (Coherent Inc., USA). The power measured in contact with the laser tip was 40 mW, whereas the optical power measured at the sample plane under the same 5-mm working distance used for irradiation was 30 mW. Therefore, all dosimetric parameters were calculated using the optical power measured at the scaffold surface. The laser handpiece had a tip diameter of 0.12 cm, corresponding to a nominal beam spot area of 0.0113 cm². Irradiation was performed with the beam positioned perpendicular to and centered on each scaffold at a fixed distance of 5 mm from the sample surface. Under this irradiation geometry, the beam covered the entire 6-mm-diameter scaffold surface. Therefore, the dosimetric parameters were calculated relative to the exposed scaffold area, which corresponded to 0.2827 cm², rather than to the nominal laser tip area.

Two PBM sessions were performed: immediately after the 24-h-cell adhesion period (T0) and again 48 h later (T48). The irradiation parameters were selected based on a previous in vitro study reporting positive effects of 660 nm PBM on SHEDs using the same radiant exposures [9], with adaptations to the scaffold-based culture model used here. The experimental groups received radiant exposures of 0.5 J/cm² (L0.5) and 1.0 J/cm² (L1), corresponding to radiant energies of 0.141 J and 0.283 J per session and exposure times of 4.7 s and 9.4 s, respectively. Each multiwell culture plate contained samples from all the experimental groups, allowing the irradiated and nonirradiated scaffolds to remain under equivalent environmental conditions during the irradiation procedure. However, only the L0.5 and L1 groups were exposed to the laser beam. Empty wells were left between seeded wells to minimize unintended light scattering. The PBM dosimetry and irradiation conditions are summarized in Table 1.

**Table 1:** Photobiomodulation parameters and irradiation conditions used in the study

Parameter	Value	Notes
Wavelength	660 nm	—
Emission mode	Continuous wave	—
Power measured at laser tip	40 mW	Measured in contact with the power meter
Optical power at sample plane	30 mW	Measured at the 5-mm working distance and used for dosimetric calculations
Laser handpiece tip diameter	0.12 cm	Nominal tip diameter
Nominal beam spot area	0.0113 cm²	Calculated from the tip diameter
Working distance	5 mm	Fixed distance from the scaffold surface
Scaffold diameter	6 mm	Circular disc
Exposed scaffold surface area	0.2827 cm²	Area used for dosimetric calculations
Beam coverage at sample plane	Whole scaffold surface	Confirmed under the 5-mm working distance
Number of irradiation points	1	Central irradiation per scaffold
Average irradiance relative to scaffold area	106.1 mW/cm²	Calculated as power/exposed scaffold surface area
Radiant exposure per session	0.5 and 1 J/cm²	Calculated relative to exposed scaffold area
Radiant energy per session	0.141 and 0.283 J	Calculated as radiant exposure × exposed scaffold area
Exposure time per session	4.7 and 9.4 s	Calculated as radiant energy/power
Number of sessions	2	T0 and T48
Interval between sessions	48 h	—
Total radiant energy over treatment course	0.283 and 0.566 J	Two sessions
Scattering control	Empty wells between seeded wells	—

Dosimetric parameters were calculated using the optical power measured at the sample plane under a 5-mm working distance. Radiant exposure and irradiance were calculated relative to the exposed area of each 6-mm-diameter scaffold disc because the beam covered the whole disc surface under this irradiation geometry. The values were rounded to three decimal places for energy and one decimal place for time

### Cell metabolic activity and viability assays

Cell metabolic activity was assessed using the Alamar Blue assay, whereas viable-cell distribution and cell death were qualitatively evaluated using the Live/Dead assay. Both assays were performed in four independent scaffold samples per group (*n* = 4). For Alamar Blue, at each experimental time point (24, 48, and 72 h), the culture medium was removed, and the cells were incubated with 10 µL of Alamar Blue reagent (Invitrogen, USA) diluted in 90 µL of α-MEM for 4 h. The reaction medium was then collected, and the absorbance was measured at 570 nm and 600 nm using a microplate reader (Epoch Biotek, USA). The percentage of Alamar Blue reduction was calculated according to the manufacturer’s instructions as an indirect measure of cell metabolic activity.

The Live/Dead assay was performed 72 h after the first irradiation using the Live/Dead Assay Cytotoxicity Kit for mammalian cells (Molecular Probes, Eugene, OR, USA). After the culture medium was removed and the samples were washed with PBS, they were incubated for 20 min in the dark with 2 µM calcein AM and 4 µM ethidium homodimer. Fluorescence images were acquired using a Zeiss Imager A2 microscope (Zeiss, Germany), and representative central-field images from the four independent scaffold samples in each group were used for qualitative assessment of viable-cell distribution and cell death.

### Cell morphology and distribution on the scaffolds

The morphology, distribution, and cell–biomaterial interactions on the PLA microfibrous scaffolds were evaluated by SEM. After 72 h of culture, the scaffolds were washed with PBS to remove nonadherent cells, fixed in 2.5% glutaraldehyde for 12 h, postfixed in 0.5% osmium tetroxide for 30 min, washed in distilled water, dehydrated in graded ethanol solutions, sputter-coated with gold, and examined using a Hitachi TM300 scanning electron microscope (Hitachi, Japan).

### Statistical analysis

Quantitative data from the Alamar Blue assay were analyzed using GraphPad Prism 8.0 software (GraphPad, USA). Data distribution was assessed using the Shapiro–Wilk test. Because the data did not meet the assumptions of normality, nonparametric tests were used. For intragroup comparisons over time, the Friedman test followed by Dunn’s post hoc test was applied, since the same scaffold samples were sequentially assessed at 24, 48, and 72 h. For intergroup comparisons at each time point, the Kruskal–Wallis test followed by Dunn’s post hoc test was used. Data are presented as medians and interquartile ranges. The level of statistical significance was set at *p* < 0.05.

## Results

**Fig. 1:**
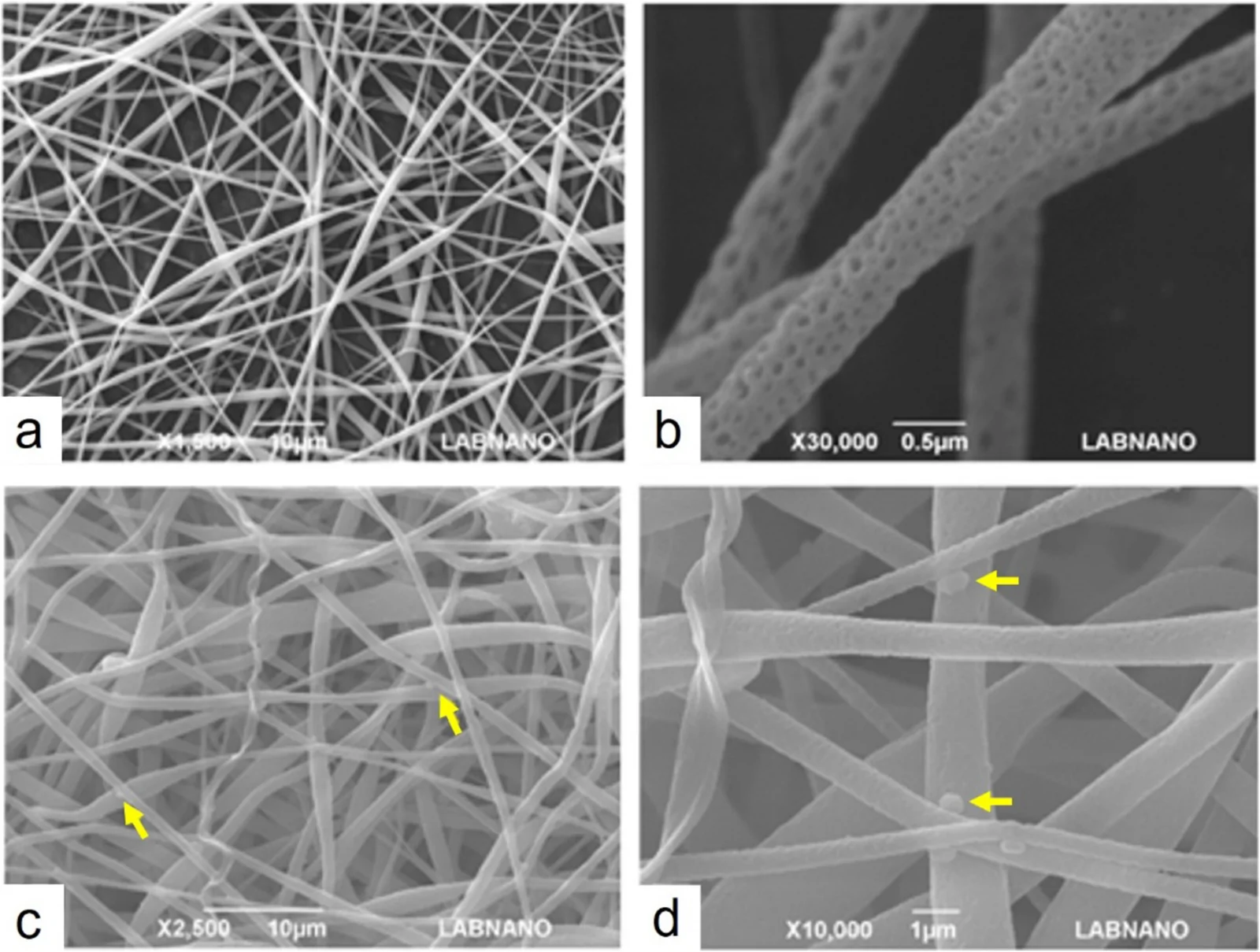
Representative scanning electron microscopy (SEM) images of electrospun PLA microfibers at different magnifications. (**a**) The scaffold exhibited a porous microfibrous architecture with randomly arranged fibers. (**b**) Higher-magnification image revealing the porous surface morphology of the fibers. (**c**, **d**) Occasional bead-like structures were observed along some fibers (yellow arrows)

SEM analysis (Fig. 1) revealed that the electrospun PLA scaffold consisted of randomly arranged microfibers that formed a porous architecture, with a mean fiber diameter of 1.87 ± 0.41 μm. At higher magnification, some fibers exhibited a porous surface morphology, and occasional bead-like structures were observed along the fibers.

**Fig. 2:**
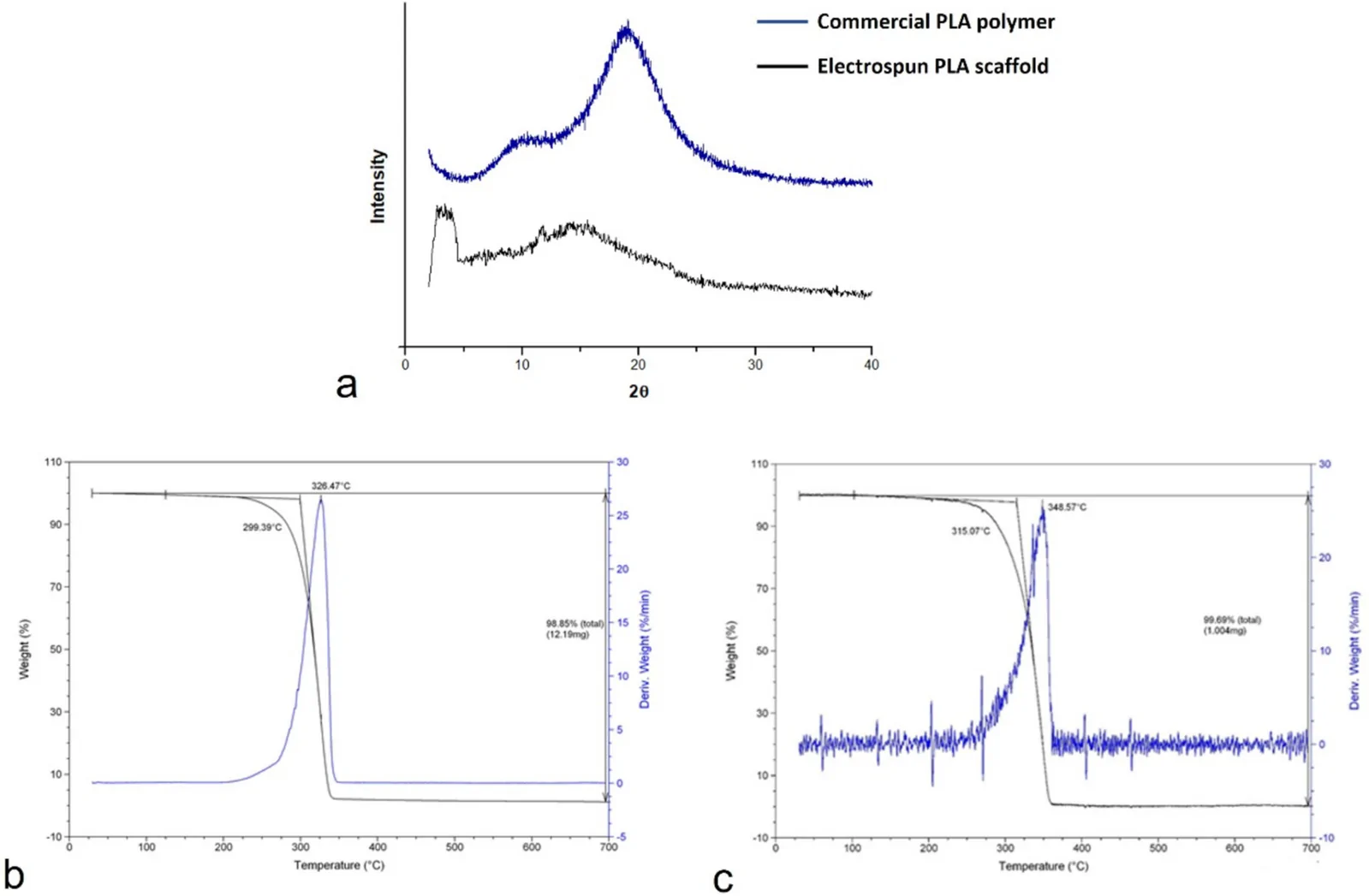
Structural and thermal characterization of the commercial PLA polymer and the electrospun PLA microfibrous scaffold. (**a**) X-ray diffraction (XRD) patterns of the commercial PLA polymer and the electrospun PLA microfibrous scaffold. (**b**, **c**) Thermogravimetric analysis (TGA) thermograms of the commercial PLA polymer and the electrospun PLA microfibrous scaffold, respectively

XRD analysis (Fig. 2a) revealed distinct diffraction profiles for the commercial PLA polymer and the electrospun PLA microfibrous scaffold. The commercial polymer exhibited more defined diffraction features, whereas the electrospun scaffold showed a broader and less intense profile, which is consistent with its lower crystallinity. Quantitative analysis based on the integrated relative peak areas revealed a degree of crystallinity of 50.29% for the commercial PLA polymer and 10.03% for the electrospun PLA microfibrous scaffold.

TGA thermograms (Fig. 2b, c) show the thermal degradation profiles of the commercial PLA polymer and the electrospun PLA microfibrous scaffold. The onset degradation temperature (Tonset) of the commercial PLA polymer was 299 °C, and the maximum degradation temperature was 326 °C (Fig. 2b). In contrast, the electrospun scaffold had a Tonset of 315 °C and a maximum degradation temperature of 349 °C (Fig. 2c). Thermal transitions, including the glass transition temperature (Tg), cold crystallization temperature (Tcc), and melting temperature (Tm), were further evaluated by DSC. The corresponding thermal properties of the commercial PLA polymer and the electrospun PLA microfibrous scaffold are summarized in Table 2.

**Table 2:** Glass transition temperature (Tg), cold crystallization temperature (Tcc), and melting temperature (Tm) of the commercial PLA polymer and the electrospun PLA microfibrous scaffold, as determined by DSC

Material	Tg (°C)	Tcc (°C)	Tm (°C)
Commercial PLA polymer	54.29	115.89	154.09
Electrospun PLA microfibrous scaffold	55.13	123.67	149.50

**Fig. 3:**
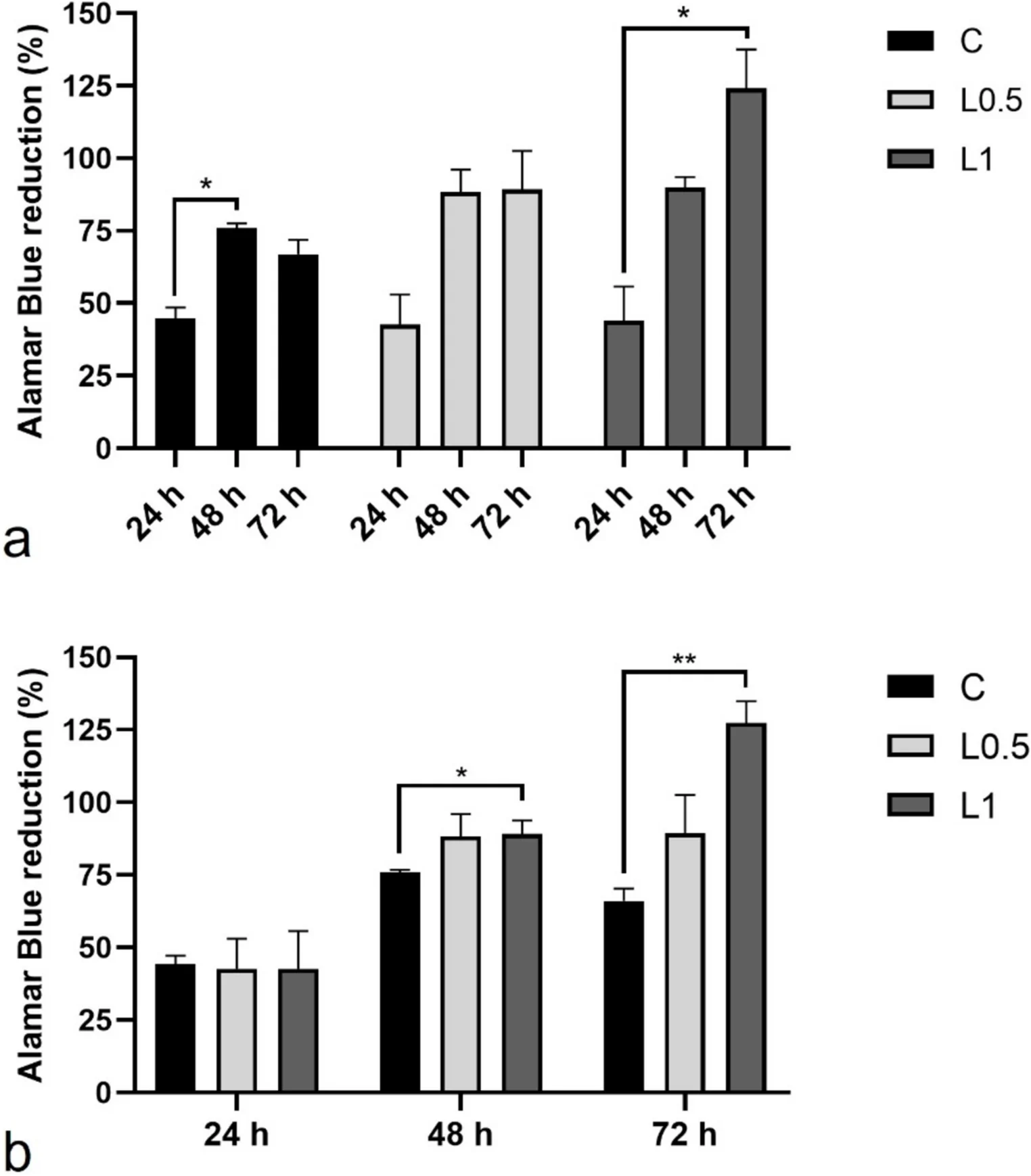
Percentage of Alamar Blue reduction in SHEDs cultured on the PLA microfibrous scaffold at 24, 48, and 72 h after the first irradiation. (**a**) Intragroup comparison of metabolic activity over time in the nonirradiated control group (C) and in the groups irradiated with 0.5 J/cm² (L0.5) or 1.0 J/cm² (L1). (**b**) Intergroup comparisons of metabolic activity among C, L0.5, and L1 at each time point. The bars represent medians, and the error bars represent the interquartile range. Intragroup comparisons over time were performed using the Friedman test followed by Dunn’s post hoc test. Intergroup comparisons at each time point were performed using the Kruskal–Wallis test followed by Dunn’s post hoc test. *p < 0.05; **p < 0.01

The results of the Alamar Blue assay revealed an overall increase in SHED metabolic activity over time in all the experimental groups cultured on the PLA microfibrous scaffold (Fig. 3a), suggesting that the scaffold supported short-term SHED culture. Intragroup analysis revealed significantly higher metabolic activity at 48 h than at 24 h in the control group (adjusted *p* = 0.0140) and at 72 h than at 24 h in the L1 group (adjusted *p* = 0.0140). No significant intragroup differences were observed in the L0.5 group. Intergroup analysis revealed no significant differences among the groups at 24 h, whereas compared with the nonirradiated control group, the L1 group presented significantly greater Alamar blue reduction values at 48 h (adjusted *p* = 0.0427) and 72 h (adjusted *p* = 0.0051) (Fig. 3b). The L0.5 group showed intermediate values at 48 and 72 h, but no statistically significant difference from the control group was detected at these time points. These findings indicate that PBM at 1.0 J/cm² increased SHED metabolic activity on the PLA microfibrous scaffold under the tested conditions, particularly at 72 h.

**Fig. 4:**
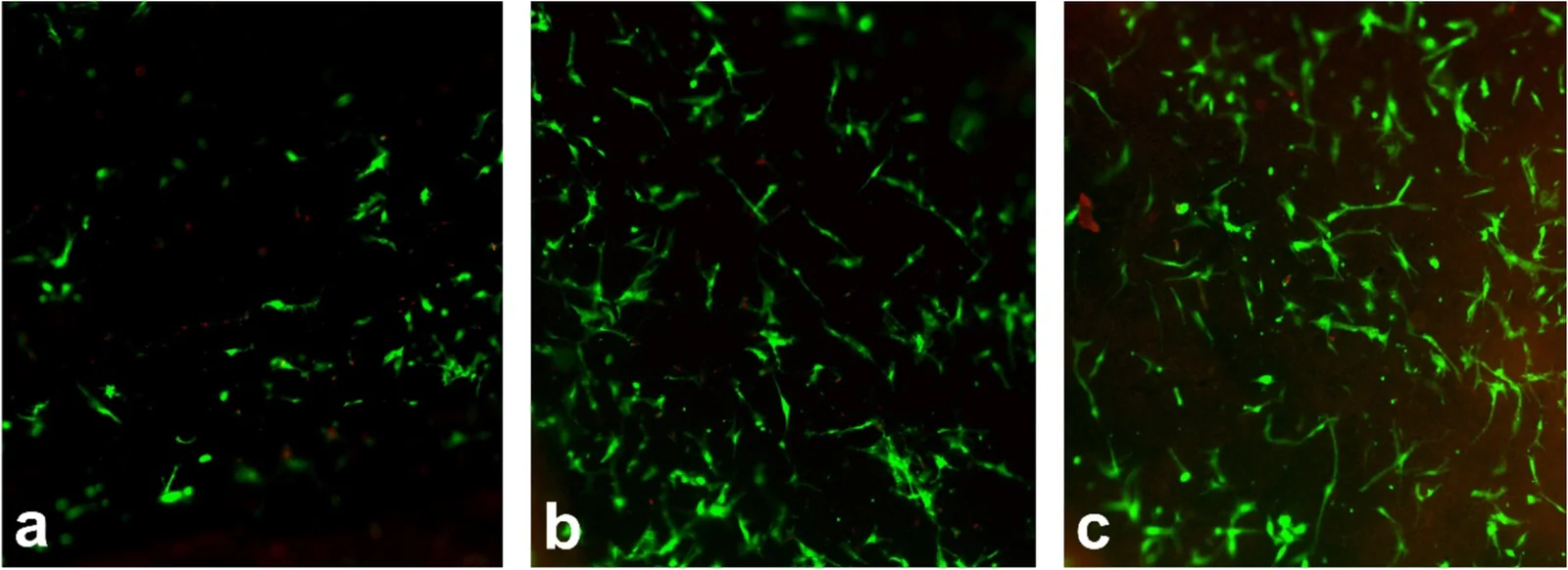
Representative Live/Dead images of SHEDs cultured on PLA microfibrous scaffolds at 72 h. (**a**) Nonirradiated control group (C); (**b**) group irradiated at 0.5 J/cm² (L0.5); (**c**) group irradiated at 1.0 J/cm² (L1). Viable cells are shown by green cytoplasmic staining with calcein AM, whereas dead cells are stained red. The irradiated groups showed qualitatively more extensive viable-cell colonization of the scaffold surface, whereas dead cells were infrequent in all groups. Scale bar = 100 μm

Representative Live/Dead images obtained at 72 h showed viable SHEDs, identified by green cytoplasmic staining with calcein AM, in all the experimental groups (Fig. 4). Compared with the nonirradiated control group, the irradiated groups showed qualitatively more extensive viable-cell colonization of the scaffold surface. Red-stained dead cells were infrequent and showed no apparent qualitative difference among the groups. These findings support the interpretation that PBM was associated with more extensive viable-cell colonization in qualitative imaging, without visual evidence of increased cell death.

**Fig. 5:**
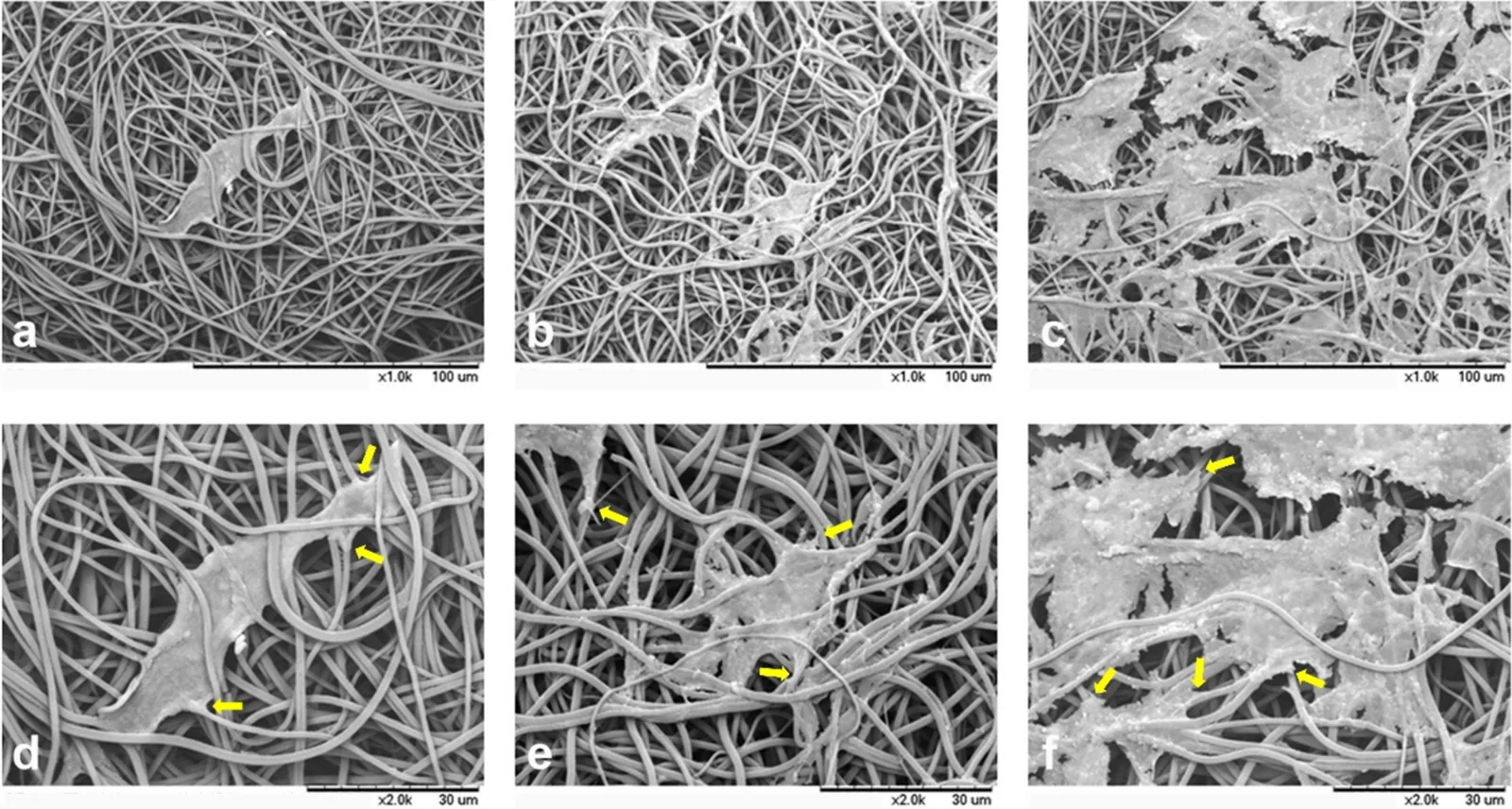
Representative scanning electron microscopy images of SHEDs cultured on the PLA microfibrous scaffold at 72 h in the nonirradiated control group (**a**, **d**), L0.5 group (**b**, **e**), and L1 group (**c**, **f**). SHEDs exhibited a fibroblast-like morphology, with cytoplasmic extensions anchored to the microfibers (arrows). The irradiated groups showed qualitatively more extensive cell colonization and coverage of the scaffold surface, particularly in the L1 group. Top row: images acquired at ×1000; scale bars = 100 μm. Bottom row: images acquired at ×2000; scale bars = 30 μm

SEM analysis performed at 72 h revealed SHED adhesion to the PLA microfibrous scaffold in all groups (Fig. 5). The cells displayed a fibroblast-like morphology, with cytoplasmic extensions anchored to the microfibers. Because of the random fiber arrangement, the cells were oriented in multiple directions. Qualitatively, compared with the nonirradiated control group, the irradiated groups showed more extensive cell colonization and coverage of the scaffold surface, particularly in L1. In this group, cells were observed not only on the surface but also among the microfibers, suggesting close interaction with the scaffold microarchitecture.

## Discussion

This study revealed that PBM with a 660 nm diode laser, particularly at 1.0 J/cm², increased short-term metabolic activity and was associated with qualitatively more extensive viable-cell colonization and favorable cell–scaffold interactions in SHEDs cultured on electrospun PLA microfibrous scaffolds. In tissue engineering, scaffold selection is important because cellular behavior is strongly influenced by the physical and chemical properties of the culture environment [19]. PLA was selected because it is widely used in biomedical applications and is known for its biocompatibility, biodegradability, and relatively low cost [6], making it suitable for tissue engineering approaches [7, 20, 21].

The versatility of PLA allows the production of microfibers by electrospinning, generating fibrous architectures that resemble fibrillar components of the native extracellular matrix (ECM), a feature associated with favorable cell–matrix interactions and signaling related to cell adhesion and behavior [22]. In the present study, SEM showed that the electrospinning parameters produced a porous PLA microfibrous scaffold with fibers distributed within a micrometric size range. Compared with traditional melt-spun fibers, electrospinning yields finer fibers with greater surface availability, which may favor cell–material interactions [23].

Some heterogeneity in fiber diameter, including thinner fibers, was also observed. This variability may reflect minor instabilities during electrospinning that affect jet stretching and fiber morphology. Occasional spherical structures on the fiber surface may likewise be related to the low viscosity of the polymer solution and droplet formation during processing [24]. In addition, the porous surface morphology observed here is consistent with the behavior of hydrophobic polymers processed under humid conditions, in which moisture-induced phase separation during solvent evaporation can contribute to pore formation [23, 25]. Because fiber production occurred under uncontrolled environmental conditions and relatively high humidity (71%), these factors likely contributed to the observed morphological pattern [4, 23, 26]. Such features may also contribute to a topography that more closely resembles the native ECM and potentially favors cell–material interactions [27].

These findings are consistent with studies indicating that electrospun PLA-based membranes combine high specific surface area, low relative density, biodegradability, and biocompatibility, making them attractive for tissue engineering applications [28]. The versatility of PLA is also linked to the possibility of modulating its physical, thermal, and mechanical behavior through composition and processing, which supports its broad biomedical use [29]. More broadly, the literature shows that electrospun fiber-based microarchitectures can actively influence stem cell behavior beyond providing mechanical support, since parameters such as fiber diameter, alignment, and surface topography affect adhesion, viability, expansion, and differentiation [30, 31]. The favorable interaction observed here between SHEDs and the PLA microfibrous scaffold may therefore reflect not only material biocompatibility but also structural cues provided by the electrospun substrate [8]. This interpretation is supported by recent evidence showing that electrospun PLA meshes can promote cell adhesion, proliferation, and organized tissue-like growth, particularly when surface properties are optimized to improve cell–material interactions [21].

Thermal characterization revealed differences between the commercial PLA polymer and the electrospun scaffold, but overall, the results indicated that electrospinning did not compromise material stability. The TGA values remained within the range reported for PLA degradation [32]. Likewise, DSC showed Tg and Tm values within the expected range for pure PLA [33]. XRD, however, revealed lower crystallinity in the electrospun scaffold than in the commercial PLA pellets, likely because of rapid solvent evaporation during jet formation, which may limit polymer chain organization. Despite this reduction, the scaffold remained semicrystalline. Since crystallinity can affect degradation behavior and cell–material interactions, this less crystalline profile may be advantageous for in vitro cell culture, especially compared with PLA films formed under slower solvent evaporation conditions [17].

Previous studies also support the biocompatibility of PLA as a substrate for stem cell-based approaches. Marei et al. [20] reported that electrospun PLA scaffolds supported the adhesion and proliferation of mesenchymal stem cells derived from bone marrow and adipose tissue, whereas Russo et al. [34] reported enhanced adhesion and proliferation of amniotic epithelial stem cells on PLA scaffolds. In addition, Wang et al. [35] reported that PLA extract did not negatively affect SHED proliferation. These findings are consistent with our Alamar Blue results, in which even the nonirradiated control group showed a progressive increase in metabolic activity over time, supporting the biocompatibility of PLA with SHEDs.

One of the main challenges in tissue engineering is obtaining an adequate number of stem cells, as their in vitro expansion is often limited [36]. In this context, PBM has emerged as a valuable strategy for enhancing stem cell biological activity and proliferation over short culture periods [16]. Positive biostimulatory effects of low-level laser irradiation have been demonstrated in stem cells of oral origin [11], as well as in various cell types cultured on biomaterials, including polycarbonate urethane [37], biosilicate [38, 39], acellular dermal matrix [40], and PLA films [13, 14]. In the present study, SHEDs cultured on PLA microfibrous scaffolds exhibited increased metabolic activity after irradiation at 1.0 J/cm², which is consistent with a positive biostimulatory effect in this in vitro model.

The positive effects of PBM on SHEDs under standard culture conditions have been previously reported [9, 41, 42] and have been associated with increased mitochondrial activity, increased ATP production, and transient reactive oxygen species generation [15]. These events are thought to contribute to the regulation of proliferation, viability, differentiation, and apoptosis through the modulation of intracellular signaling pathways and gene expression [12, 17]. In agreement with this rationale, the present study revealed that irradiation at 1.0 J/cm² increased SHED metabolic activity, whereas Live/Dead imaging suggested qualitatively more extensive viable cell colonization of the scaffold surface in the irradiated groups, without visual evidence of increased cell death.

Our findings are also consistent with those of previous studies evaluating PBM in biomaterial-supported systems. Choi et al. [40] reported positive effects of 1 J/cm² irradiation on mesenchymal cells cultured on an acellular dermal matrix, and Hsu et al. [37] reported improved responses in endothelial cells cultured on a porous polyurethane scaffold after irradiation at 1.18 J/cm². Although irradiation in the latter study was applied before cell seeding because of scaffold porosity, both studies support the interpretation that PBM can enhance cell behavior in the presence of biomaterials. In the present study, direct irradiation after cell adhesion also resulted in increased metabolic activity, supporting the potential of PBM as an adjunctive biophysical stimulus for early cell–scaffold responses in tissue engineering models.

Previous studies have shown that cell responses are not always favorable when higher PBM doses are used. Renno et al. [38] reported reduced proliferation of MC3T3-E1 preosteoblastic cells cultured on biosilicate after irradiation at 10 J/cm², and Pinto et al. [39] reported no additional regenerative effects when a biosilicate scaffold was combined with 120 J/cm² laser therapy in a rat bone defect model. These findings may reflect interactions between the laser and the biomaterial, such as light refraction or absorption, which can affect effective energy delivery [38, 39]. More broadly, PBM responses are highly dependent on the optimization of wavelength, irradiance, energy density, and exposure time and commonly follow a biphasic dose‒response pattern, in which suboptimal or excessive doses may reduce or abolish stimulation [12]. In contrast, the PLA microfibrous scaffold used here did not appear to prevent the response to low-dose irradiation, since the metabolic activity of the 1.0 J/cm² group was greater than that of the control group at 48 and 72 h. The 0.5 J/cm² group showed intermediate values but did not differ significantly from the control group according to the results of the present statistical analysis. In support of this interpretation, Sabino et al. [13] and Lira et al. [14] reported beneficial effects of low-dose 660 nm PBM on cells cultured on PLA-based substrates.

Additional evidence from polymer-based scaffolds and three-dimensional culture systems further supports the interpretation that PBM can exert favorable effects at the cell–material interface. Positive responses, including increased viability and improved cell–material interactions after irradiation, have been reported in periodontal-related stem cells cultured on PLA-based substrates [14]. Similarly, studies using three-dimensional culture systems have indicated that PBM may increase metabolic activity and other biologically relevant responses under biomaterial-supported conditions [43]. Although direct evidence specifically involving SHEDs cultured on electrospun PLA microfibrous scaffolds remains limited, the available literature supports the interpretation that PBM may function as a useful adjunctive biophysical stimulus in this context.

Recent studies have further reinforced this concept. Hany et al. [44] reported that an infrared diode laser combined with nanofibrous polycaprolactone/alginate scaffolds enhanced the proliferative and osteogenic responses of gingival mesenchymal stem cells. Similarly, Abo El-Dahab et al. [45] reported enhanced attachment, proliferation, and osteogenic commitment of human periodontal ligament stem cells cultured on titanium implants after irradiation with an infrared diode laser. Together, these studies support the relevance of PBM as an adjunctive tool in regenerative and implant-related applications.

Despite these promising findings, the results of the present study should be interpreted within its short-term in vitro design. The analyses focused on early SHED responses up to 72 h, particularly metabolic activity, viable-cell distribution, and cell–scaffold interactions. Therefore, longer-term studies using complementary quantitative and functional assays are needed to determine whether these early responses are sustained and whether they influence later events relevant to tissue engineering. In addition, although the irradiation geometry ensured macroscopic coverage of the whole 6-mm scaffold surface, detailed beam profile analysis and light distribution mapping within the microfibrous scaffold were not performed; therefore, local variations in light delivery within the scaffold architecture cannot be excluded.

Future studies should expand these findings by evaluating additional PBM parameters, scaffold designs, and longer culture periods. Analyses involving quantitative cell proliferation, osteogenic/odontogenic differentiation, extracellular matrix formation, and mineralization would help determine whether the early PBM-associated responses observed here translate into improved tissue engineering performance. Studies including SHEDs from different donors and in vivo models may also broaden the biological and translational relevance of this approach.

## Conclusion

The results of the present study revealed that electrospun PLA microfibrous scaffolds supported short-term SHED culture and that PBM at 1.0 J/cm², under the tested irradiation conditions, increased SHED metabolic activity and was associated with favorable qualitative cell–scaffold interactions at 72 h. These findings provide preliminary evidence that PBM may serve as a useful adjunctive biophysical stimulus during the early culture of SHEDs on PLA-based microfibrous scaffolds.

## Data Availability

The datasets generated and/or analyzed during the current study are available from the corresponding author on reasonable request.

## Abbreviations

a-MEM: Alpha Minimum Essential Medium
ATP: Adenosine Triphosphate
BMP-2: Bone Morphogenetic Protein-2
CD: Cluster of Differentiation
CO₂: Carbon Dioxide
DMSO: Dimethyl Sulfoxide
DMF: Dimethylformamide
DSC: Differential Scanning Calorimetry
ECM: Extracellular Matrix
FBS: Fetal Bovine Serum
HLA-DR: Human Leukocyte Antigen – DR isotype
J/cm²: Joules per Square Centimeter
L0.5: Photobiomodulation group irradiated with 0.5 J/cm²
L1: Photobiomodulation group irradiated with 1.0 J/cm²
PBM: Photobiomodulation
PBS: Phosphate-Buffered Saline
PLA: Polylactic Acid
SEM: Scanning Electron Microscopy
SHEDs: Stem Cells from Human Exfoliated Deciduous Teeth
T0: First irradiation time point (immediately after adhesion period)
T48: Second irradiation time point (48 h after T0)
TGA: Thermogravimetric Analysis
Tg: Glass Transition Temperature
Tcc: Cold Crystallization Temperature
Tm: Melting Temperature
XRD: X-ray Diffraction
